# The challenges of hand hygiene improvement: a comparison between inpatient and outpatient units

**DOI:** 10.1186/1753-6561-5-S6-P110

**Published:** 2011-06-29

**Authors:** JY Kawagoe, CV Silva, MF Cardoso, P Gonçalves, MG Ballalai, AR Toniolo, CB DalForno, ST Valerio, EAA Reis, LG Pontes, LB Cunha, L Correa

**Affiliations:** 1 Infection Prevention and Control Service, São Paulo, Brazil; 2 Education and Research Institute, São Paulo, Brazil; 3 Patient Safety, Hospital Albert Einstein, São Paulo, Brazil

## Introduction / objectives

WHO Hand Hygiene (HH) Improvement Strategy Program has been implemented mostly in inpatient units. We implemented this strategy in inpatient and outpatient units.

## Objective

Compare the results of HH adherence in inpatient and outpatient units.

## Methods

This program was conducted in a 500-bed private hospital in São Paulo, Brazil. WHO tool was used to assess HH adherence before (Jun-Aug2008) and after the intervention (Jul-Sept2009). The inpatient units were: Medical Surgical Ward, Oncology, Neonatal and Pediatrics and outpatient were: Surgical Ambulatory, Obstetric Center, Emergency, Post Anesthesia, Diagnostic Medicine Department and Dialysis Center. Several strategies were applied – campaigns, behavioral methods, and educational programs. More dispensers of alcohol-based product were made available in areas of the hospital such as restaurants, cafeterias, waiting rooms, lounges, receptions and next to elevators.

## Results

In inpatient units the overall HH adherence improved 30.8%, from 63.8% (1433/2248 opportunities) to 83.5% (2144/2567 opportunities). In outpatient units it improved 53.2%, from 39.5 % (679/1721 opportunities) to 60.4% (1044/1728 opportunities). The only exception in HH improvement was physician (no increase in inpatient unit) and Obstetric Center (5% decrease).

## Conclusion

The higher compliance was in inpatient units, although there was a significant improvement in outpatient units. We must develop and apply different strategies according to the needs of inpatient and outpatient units.

## Disclosure of interest

None declared.

